# The impact of a high‐fat diet on liver health in pregnant mice and their offspring: The role of the gut‐liver axis

**DOI:** 10.1002/imo2.70026

**Published:** 2025-06-03

**Authors:** Qian Gong, Yufei Zhang, Juxiong Liu, Shuai Yuan, Huijie Hu, Yu Cao, Shoupeng Fu, Wenjin Guo

**Affiliations:** ^1^ State Key Laboratory for Diagnosis and Treatment of Severe Zoonotic Infectious Diseases, Key Laboratory for Zoonosis Research of the Ministry of Education, Institute of Zoonosis, and College of Veterinary Medicine Jilin University Changchun China; ^2^ Chongqing Research Institute Jilin University Chongqing China; ^3^ Department of Veterinary Medicine, College of Animal Sciences Zhejiang University Hangzhou China

## Abstract

- High‐fat diet during pregnancy induced hepatic steatosis.
- High‐fat diet during pregnancy led to altered gut microbiota and metabolic disruptions.
- High‐fat diet during pregnancy disrupted the intestinal metabolites such as lithocholic acid (LCA).
- LCA altered in high‐fat diet induced hepatic inflammation both in pregnant mice and their offspring.

## Graphical abstract

The study investigated the longitudinal effects of normal and high‐fat diet, showing their differential effects on gut microbiota diversity and gut metabolites, with the latter having a significant impact on increasing liver inflammation and gut microbiota and metabolites disruption.

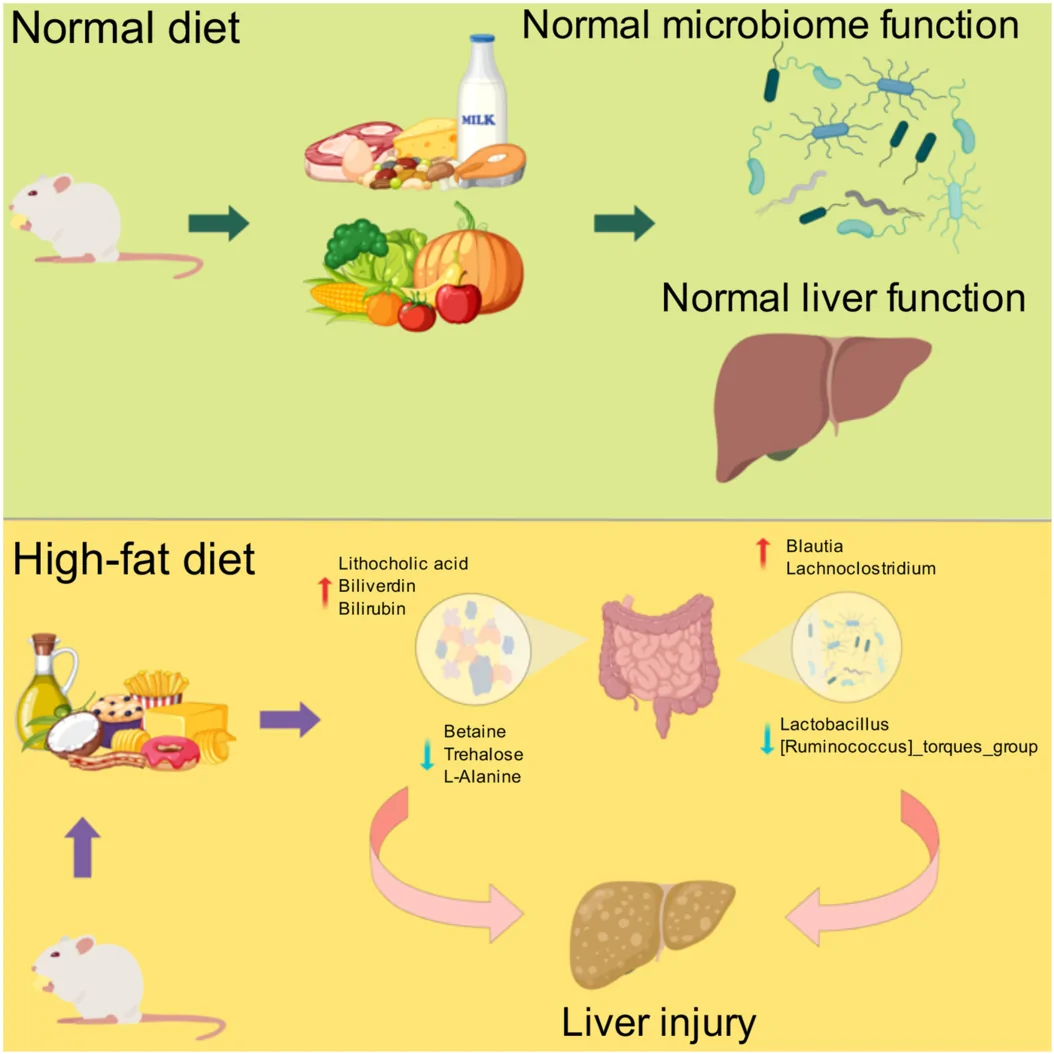

To the editor,

High‐fat diet (HFD) has long been known to impact the intestinal microbiome and metabolism. During pregnancy, the mother's gut microbiota can influence the growth and development of the fetus. Disruption in the metabolic products synthesized by maternal gut bacteria could also result in disease in the offspring. While researchers have primarily focused on the impact of HFD on fetal growth, few studies have examined whether HFD affects the mother during pregnancy. Our study demonstrates that HFD during pregnancy is strongly associated with disruptions in the gut microbiota, often leading to disturbances in intestinal metabolites, notably bile acid metabolism. When bile acids were fed to normal pregnant mice, both the pregnant mice and their offspring showed signs of liver damage. This suggests that the changes in the gut microbiota and the metabolic disruptions caused by HFD not only result in liver damage in the mother but can also affect the offspring. These findings underscore the importance of a balanced diet for pregnant women.

## 1 RESULTS AND DISCUSSION

High‐fat diet (HFD) induces liver injury and change of gut microbiota composition

In recent times, dietary lipid intake has increased with improved living standards, resulting in fat accumulation and nonalcoholic fatty liver disease (NAFLD). HFD could alter gut microbiology with a corresponding change in gut metabolites. However, little attention has been given to alterations in gut microbiota and metabolites due to HFD among pregnant women. Thus, we examined the effects of HFD during pregnancy on pregnant female mice.

**FIGURE 1:**
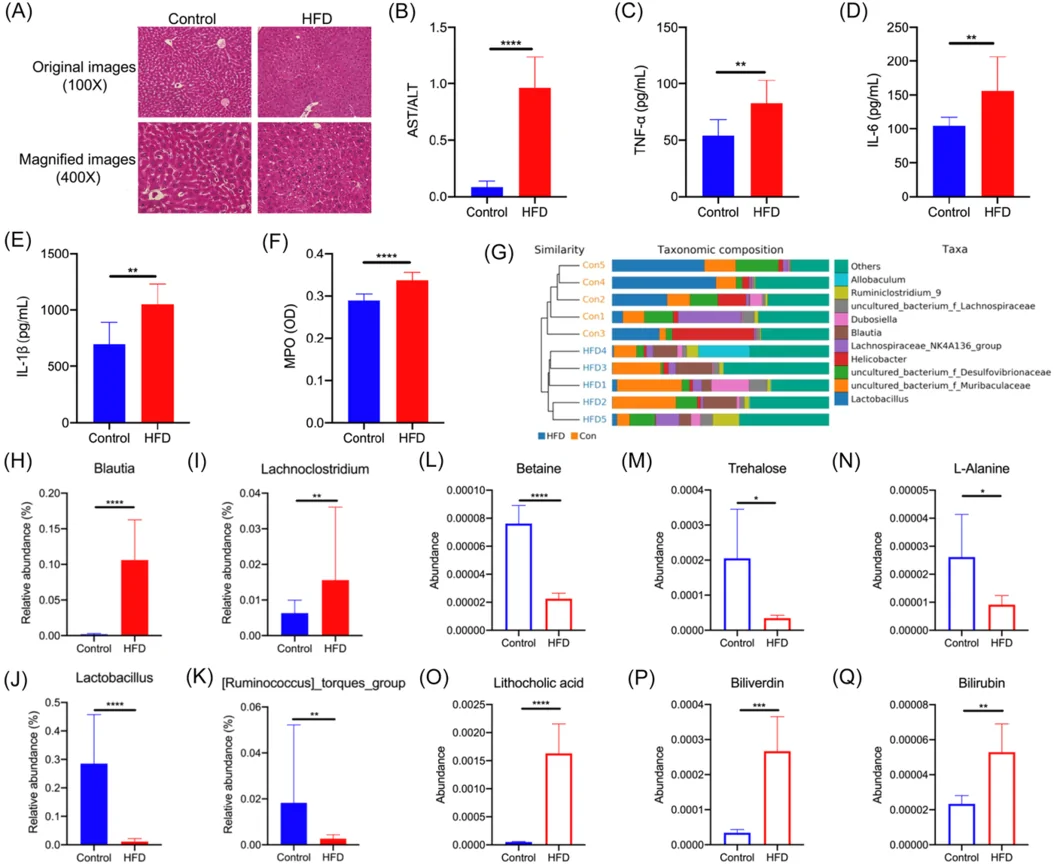
High‐fat diet (HFD) induces liver injury and change of gut microbiota composition. (A) Hematoxylin and eosin (H&E) staining of liver tissues from control and HFD groups. (B) Liver function tests: aspartate aminotransferase (AST), alanine aminotransferase (ALT), and the ratio between them are used to assess liver function and injury (*n* = 3). (C–E) The expression levels of pro‐inflammatory mediators, such as tumor necrosis factor alpha (TNF‐α), interleukin‐6 (IL‐6), and interleukin‐1β (IL‐1β), were assessed in liver samples by ELISA (*n* = 3). (F) Myeloperoxidase (MPO) content in liver tissue was measured (*n* = 3). (G) Unweighted pair‐group method with arithmetic means (UPGMA) clustering tree histogram. The shorter the branch lengths, the greater the similarity in species composition between samples. The abundance histogram illustrates species diversity, abundance similarity, and dominant species across samples based on the proportion of each color block. (H–K) Histogram showing abundance of marker bacteria between groups (*n* = 5). (L–Q) Representative differential metabolite abundance statistics plot (*n* = 5). Data are means ± standard deviation (SD). *T*‐test was used to analyze the difference between the control group and HFD group. *p < 0.05, **p < 0.01, ***p < 0.001, ****p < 0.0001.

HFD has been well‐documented to induce hepatic steatosis, a condition characterized by excessive fat deposition within hepatocytes that progresses to liver disease in the absence of overt hepatocellular swelling [1]. The accumulation of lipid droplets leads to lipid toxicity and the release of damage‐associated molecular patterns, activating Kupffer cells and promoting inflammation [2]. This pathological process was confirmed by hematoxylin and eosin staining (Figure 1A), which demonstrated notable neutrophil presence and significant hepatocyte degeneration in the liver tissue of the HFD group. Concurrently, alterations in alanine aminotransferase (ALT) and aspartate aminotransferase (AST), key indicators for assessing liver function, aligned with the observed liver cell damage (Figure 1B). Our study also revealed that maternal HFD consumption during pregnancy increases hepatic inflammation and markedly increases the expression of pro‐inflammatory cytokines including tumor necrosis factor alpha (TNF‐α), interleukin‐1β (IL‐1β), and interleukin‐6 (IL‐6) in liver tissue (Figure 1C–E), along with myeloperoxidase (MPO), a peroxidase heavily secreted by activated neutrophils (Figure 1F). Collectively, these findings suggest that HFD can cause liver damage through two mechanisms: by promoting fat accumulation in organs like the liver, leading to liver inflammation and a greater metabolic burden, and by disrupting the composition of the gut microbiota, thereby releasing harmful metabolites that can damage the liver.

We investigated the expression levels of proteins related to energy metabolism and fatty acid synthesis, which were significantly altered after HFD treatment. Specifically, the level of phosphorylated ACC decreased significantly after HFD (Figure S1A,B), indicating metabolic disorder in the liver. Additionally, fatty acid synthesis‐related proteins FASN and SRBP1 were significantly elevated in the HFD model (Figure S1A,C,D), suggesting that HFD damages the livers of pregnant mice by mediating energy metabolism and fatty acid synthesis. To explore the mechanism of HFD‐induced hepatic inflammation, we assessed the activation of the NF‐κB signaling pathway. Our results showed that HFD induced NF‐κB p65 phosphorylation (Figure S1A,E), along with elevated p‐p38 levels in HFD model mice (Figure S1A,F). These findings *suggested* that HFD promotes the phosphorylation of MAPK and NF‐κB signaling pathways, ultimately inducing hepatic inflammation.

The gut‐liver axis, a critical communication pathway, governs interactions between the intestinal milieu and hepatic function [3]. The liver provides bile salts and antibacterial compounds to the intestine via the bile duct, thereby regulating microbial growth and maintaining gut microbiota balance. Conversely, gut‐derived substances, including inflammatory mediators and butyric acid, influence hepatic function and modulate the intestinal barrier's integrity [4]. Perturbations in this cyclical relationship, such as gut dysbiosis, can perpetuate chronic low‐grade inflammation, worsening conditions like HFD‐induced liver disease [5]. To investigate the effects of HFD during pregnancy, we analyzed gut microbiota alterations. We utilized 16S rDNA high‐throughput sequencing to analyze the gut microbiota composition of HFD mice. By examining the Shannon index curves (Figure S2A) and genus‐level species accumulation plots (Figure S2B), we confirmed that the control and HFD group samples met the basic requirements for data analysis. The pCoA analysis indicated significant differences between the two groups of samples (Figure S2C). UPGMA analysis (Figure S2D) and heat map (Figure S2E) of sample clustering showed similar results. Using clustering, we analyzed the enriched flora from the control and HFD groups, which revealed a significant overlap between them (Figure S2F). This suggested that shared microorganisms did not significantly change in different environments; however, HFD caused some flora imbalance. By utilizing UPGMA clustering trees and histograms (Figure 1G), we observed that HFD induction significantly reduces the proportion of *Lactobacillus* and *Helicobacter*, while increasing the proportion of *Muribaculaceae* and *Blautia* in the intestine. These results align with previous studies [6, 7]. HFD‐induced liver inflammation is a type of NAFLD, where *Lachnospiraceae* is often more abundant [8]. As beneficial bacteria in the gut microbiota, *Lactobacillaceae* can inhibit inflammatory factors such as IL‐1β, IL‐6, and TNF‐α by producing compounds that deter pathogenic bacteria and promote a healthier gut microbiome [9]. To gain a detailed understanding of gut microbe changes under HFD conditions, we plotted abundance histograms between marker groups (Figure 1H–K). In terms of elevated flora in the gut, representative species include *Blautia* and *Lachnoclostridium*. Furthermore, we analyzed gut microbiota and classified them into aerobic and anaerobic, gram‐positive, and gram‐negative bacteria (Figure S3A–D). Results showed that certain groups of bacteria were highly specific to particular groups, and HFD treatment was associated with an increase in anaerobic bacteria and a decrease in aerobic bacteria. In summary, HFD may cause imbalances in the gut microbiota, reducing beneficial bacteria and promoting harmful bacteria. This leads to a greater risk of NAFLD and other metabolic diseases in pregnant women, emphasizing the need for a balanced diet during pregnancy.

An imbalance of gut microbiota inevitably affects metabolite differences, and thus, we utilized untargeted metabolomics to analyze the differences in metabolites in the gut of normal and HFD‐fed pregnant mice. Initially, we conducted a comprehensive analysis of all the metabolites obtained from our screening process. The outcome revealed an evident separation between the control and HFD groups, with noticeable subgroups formed among the metabolites of different groups (Figure S4A,B). Additionally, the assessment of duplicate sample correlation demonstrated a high level of correlation within different groups, but considerable variability existed between various groups (Figure S4C). With this in mind, these results suggested that the collected samples are robustly valid. Subsequently, we performed further analysis on the differential metabolites to ascertain more specific outcomes. PCA showed that the control and HFD groups exhibited a significant split (Figure S4D). The differential metabolism clustering heat map clearly showed the differences in various metabolites between different groups (Figure S4E). We screened a total of 650 differential metabolites in this study, of which 281 were upregulated and 369 were downregulated shown in Figure S4F. After annotating the screened differential metabolites, we found that most of the differential metabolites clustered among carboxylic acids and derivatives, organooxygen compounds, and steroids and steroid derivatives (Figure S4G). KEGG results of the differential metabolites revealed associations with a variety of metabolic pathways, including steroid biosynthesis, ubiquinone and other terpenoid‐quinone biosynthesis, amino sugar and nucleotide sugar metabolism, regulation of lipolysis in adipocytes, and galactose metabolism (Figure S4H).

Bile salts play a pivotal role in regulation of various hepatic metabolic processes, including bile acid synthesis, glucose metabolism, and lipid metabolism. To understand the impact of HFD during pregnancy, we used intestinal microbial metabolomics to examine the effects on intestinal microbial metabolites (Figure 1L–Q). Levels of bilirubin and biliverdin, important metabolites markers, were significantly elevated in response to the consumption of HFD. Elevated bilirubin levels suggest hepatocyte stress or swelling, resulting in intrahepatic bile duct compression and impaired bile flow [10]. Biliverdin, known for its anti‐inflammatory properties, offers protection against various diseases like vascular injury, suggesting potential intestinal self‐protection after HFD treatment [11]. In contrast, betaine, a metabolite that helps lower blood glucose, decreased significantly after HFD treatment, indicating a potential increase in the prevalence of diabetes during pregnancy [12]. Bile acids in the enterohepatic circulatory system are critical for fat metabolism [13]. Gut microbiota with bile salt hydrolase activity, such as *Bifidobacterium*, *Lactobacillus*, and *Bacteroides*, play an essential role in regulating bile acid metabolism [14]. These bacteria perform deconjugation in the ileum, converting bound bile acids to unconjugated bile acids, which are then transformed into secondary bile acids, including lithocholic acid, via hydroxylation and differential isomerization [15]. Our study found that the alterations in gut microbiota and metabolite disorders interact, contributing to metabolic and inflammatory issues during pregnancy.

Lithocholic acid (LCA) induces liver injury and pro‐inflammatory mediators in pregnant mice and their offspring

**FIGURE 2:**
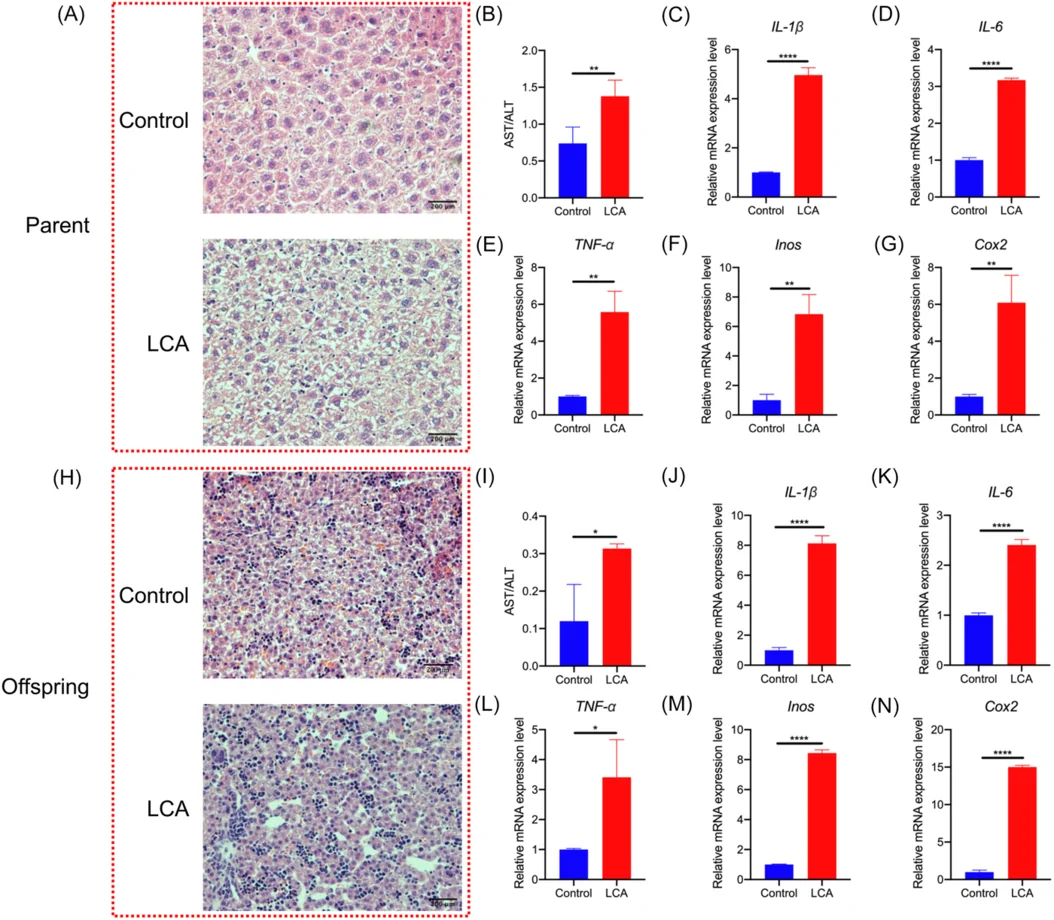
Lithocholic acid (LCA) induces liver injury and pro‐inflammatory mediators in pregnant mice and their offspring. (A) H&E staining of liver tissues from control and LCA‐treated mice. (B) Liver function tests, including AST, ALT, and their ratio, can be used to evaluate liver function or liver injury. (C–G) Expression levels of pro‐inflammatory mediators *IL‐1β*, *IL‐6*, *TNF‐α*, *Inos*, and *Cox*2 were assessed in pregnant mice's liver tissues using quantitative qRT‐PCR. (H) H&E staining of liver tissues from control and LCA‐treated mice. (I) Liver function tests, with the same measurements used for evaluation. (J–N) Expression levels of pro‐inflammatory mediators *IL*‐*1β*, *IL*‐*6*, *TNF*‐*α*, *Inos*, and *Cox*2 were assessed in the livers of offspring using qRT‐PCR. Data are presented as mean ± SD (*n* = 6). *T*‐test was used to analyze the difference between the control group and HFD group. *p < 0.05, **p < 0.01, ***p < 0.001, ****p < 0.0001.

In previous studies, we found that HFD causes significant alterations in intestinal metabolites, with LCA emerging as one of the most prominent metabolites. Bile acids and gut microbiota are inherently interconnected in a bidirectional and dynamic relationship, wherein bile acids influence gut microbial composition, and gut microorganisms, in turn, modulate both the composition and total pool of bile acids, ultimately impacting systemic metabolism [16]. To investigate whether elevated levels of LCA contribute to liver inflammation in pregnant female mice, we administered LCA orally and monitored its effects on liver health. We noted that although different from HFD‐induced fatty liver, LCA provoked hepatic inflammatory lesions characterized by neutrophilia and hepatic vacuolization (Figure 2A). qPCR was performed on liver tissue samples for inflammatory factors. Our findings have revealed a significant increase in the expression levels of inflammatory genes *IL‐1β*, *IL‐6*, *TNF‐α*, *Inos*, and *Cox2* in the liver tissues of subjects administered with LCA compared to the controls (Figure 2B–G). These outcomes further corroborate the presence of an ongoing inflammatory response in the liver, which is similar to that induced by HFD.

As LCA has been shown to cause liver inflammation in pregnant mice, it is reasonable to assume that it may also impact the liver function of their offspring. Therefore, we evaluated the hepatic function and inflammatory indexes of the offspring mice to investigate this possibility. Our findings indicate that LCA administration led to the development of liver lesions in the offspring, as evidenced by an increased infiltration of hepatic inflammatory factors, severe vacuolization, and impaired hepatic function compared to the control group (Figure 2H), consistent with our prediction. Furthermore, the levels of inflammatory genes *IL‐1β*, *IL‐6*, *TNF‐α*, *Inos*, and *Cox2* were significantly elevated in the liver tissues of the LCA group's offspring, which closely aligns with the phenotype observed in pregnant mice (Figure 2I–N). These results suggested that LCA‐related abnormalities not only lead to hepatic inflammation and compromised liver function in female mice but also extend to their offspring. These observations suggest that high levels of LCA during pregnancy can have detrimental effects not only on the mother but also on the offspring's liver health.

## 2 CONCLUSION

In summary, our study demonstrated that HFD induces significant shifts in gut microbiota composition and the production of harmful metabolites, reducing the abundance of beneficial bacteria and increasing the growth of pathogenic bacteria. The resulting metabolites can have adverse effects on the liver through the enterohepatic axis. Of particular concern is the impact of LCA on liver health, as it has the potential to induce liver inflammation in pregnant females and their offspring. Consequently, our research strongly advises against prolonged high‐fat diets during pregnancy, and suggests that modifying dietary components may improve liver health outcomes for both pregnant women and their children.

## AUTHOR CONTRIBUTIONS

**Qian Gong**: Writing—original draft; investigation; project administration. **Yufei Zhang**: Data curation; investigation. **Juxiong Liu**: Funding acquisition; writing—review and editing. **Shuai Yuan**: Validation; formal analysis; software. **Huijie Hu**: Supervision; visualization. **Yu Cao**: Supervision; formal analysis; resources. **Shoupeng Fu**: Funding acquisition; writing—review and editing. **Wenjin Guo**: Funding acquisition; writing—review and editing; conceptualization; methodology.

## CONFLICT OF INTEREST STATEMENT

The authors declare no conflicts of interest.

## ETHICS STATEMENT

This study involved animal subjects and it complies with ARRIVE guidelines. No human subjects. This study was approved by the Animal Protection and Use Committee of Jilin University, and all animal experiments were conducted in strict accordance with the guidelines of the Animal Protection and Use Committee of the institution (Permit number: SY202309040).

## Supporting information


**Figure S1.** High‐Fat Diet (HFD) Induces Metabolic Disorders and Inflammation in Liver.
**Figure S2.** The Impact of HFD on the Intestinal Microbiota.
**Figure S3.** Analysis of Gut Microbiota Composition.
**Figure S4.** Non‐Targeted Metabolomic Analysis of Mouse Gut Microbiota.
**Table S1.** Primers for qRT‐PCR.

## Data Availability

All data generated and analyzed during this study are included in this published article and its additional files. The data and scripts used are saved in Gitee: https://gitee.com/gq1022/hfd-pregnant-mouse.git. Supplementary materials (methods, figures, tables, graphical abstract, slides, videos, Chinese translated version, and update materials) may be found in the online DOI or iMeta Science http://www.imeta.science/imetaomics/. The data that support the findings of this study are available on request from the corresponding author. The data are not publicly available due to privacy or ethical restrictions.
