# Supplementary material for: The impact of a high‐fat diet on liver health in pregnant mice and their offspring: The role of the gut‐liver axis
Source: IMetaOmics. 2025 Jun 3;2(3):e70026. doi: 10.1002/imo2.70026 (PMC12806059; doi:10.1002/imo2.70026)
Supplement: Supplementary file 1 — Figure S1. High‐Fat Diet (HFD) Induces Metabolic Disorders and Inflammation in Liver. Figure S2. The Impact of HFD on the Intestinal Microbiota. Figure S3. Analysis of Gut Microbiota Composition. Figure S4. Non‐Targeted Metabolomic Analysis of Mouse Gut Microbiota. Table S1. Primers for qRT‐PCR. [file IMO2-2-e70026-s001.docx]

**Supporting information to**

**The Impact of a High-Fat Diet on Liver Health in Pregnant Mice and Their Offspring: The Role of the Gut-Liver Axis**

Running title: Gut-Liver Axis in High-Fat Diet Pregnant Mice and Their Offspring

Qian Gong^#1, 3^, Yufei Zhang^#1^, Juxiong Liu^1^, Shuai Yuan^1^, Huijie Hu^1^, Yu Cao^1^, Shoupeng Fu^1*^ and Wenjin Guo^1, 2*^

1. State Key Laboratory for Diagnosis and Treatment of Severe Zoonotic Infectious Diseases, Key Laboratory for Zoonosis Research of the Ministry of Education, Institute of Zoonosis, and College of Veterinary Medicine, Jilin University, Changchun, 130062, China

2.Chongqing Research Institute, Jilin University, Chongqing, 401120, China

3. Department of Veterinary Medicine, College of Animal Sciences, Zhejiang University, Hangzhou, 310058, China

^#^ These authors contributed equally: Qian Gong, Yufei Zhang.

^*^Correspondence: guowenjin@jlu.edu.cn (Wenjin Guo); fushoupeng@jlu.edu.cn (Shoupeng Fu).

# **Supplementary methods**

**Animal experiments**

All animal procedures adhered to established guidelines, and the research protocol was approved by the Ethics Committee of Jilin University (Approval Number: SY202309040). C57BL/6 mice (6–8 weeks old; *n* = 12) were obtained from Liaoning Changsheng Biotechnology Co., Ltd. The mice were randomly divided into two groups (*n* = 6/group). Female mice were fed the respective diets for 6-10 weeks until high-fat diet (HFD) animals gained 20% of their initial body weight [1].To establish mating, one male mouse and two female mice were caged together at 18:00, and the following morning, between 8:00 and 9:00, female mice were checked for vaginal plugs. The presence of a vaginal plug indicated successful mating and was recorded as the first day of pregnancy. Male and female mice that did not mate were returned to their respective cages and the mating process was repeated. The pregnant female mice were then divided into a control group and a high-fat diet group. The high-fat diet group (HFD, composed of 40%-fat, 20%-protein, 40%-carbohydrates based on caloric content, Xiaoshu Youtai Biological Company, China) began its regimen on the third day of pregnancy, while the control group (normal diet, composed of 12%-fat, 23%-protein, 65%-carbohydrates based on caloric content, Changsheng Biological Co., LTD., China) continued on a standard diet [2]. The mice were kept on a 12-hour light/dark cycle at 22°C, with free access to food and water. On the 18th day of pregnancy, the mice were decapitated to collect liver and fecal samples for analysis of fecal metabolomics and gut microbiota. To assess the effects of lithocholic acid (LCA), pregnant mice were separated into two groups (*n* = 6/group), with one group receiving LCA and the other serving as the control. LCA was dissolved in dimethyl sulfoxide (DMSO) at a concentration of 250 mg/ml. An emulsion was prepared by combining one part of the dissolved LCA in DMSO with nine parts of a 0.5% sodium carboxymethylcellulose solution (a 1:9 ratio). This emulsion was administered to the pregnant mice via gavage at a dose of 300 mg/kg/day until delivery. On the day of birth (pups’ day 0), the offspring from these experiments were also divided into groups (*n* = 6/group) for further study. All the mice and those offspring were anesthetized with isoflurane and decapitated in a room separate from where the other animals were kept.

**Myeloperoxidase (MPO) in liver tissue**

Fresh liver tissue was homogenized with HEPES buffer containing HEPES at a weight-to-volume ratio of 1:4 (g: mL). The homogenate was centrifuged at 13,000 rpm for 20 minutes, and the supernatant was stored at -80°C for ELISA detection. For MPO detection, the same weight-to-volume ratio was used with 0.5% hexadecyl trimethyl ammonium chloride (CTAC) in place of HEPES. After centrifugation at 13,000 rpm for 20 minutes, the supernatant was collected for MPO detection. The MPO results were quantified by optical density (OD) values.

**Enzyme Linked Immunosorbent Assay (ELISA)**

The test samples were liver homogenate supernatants stored at -80°C. The experimental procedures for ELISA followed the standard MPO detection protocol [3].

**Alanine Transaminase (ALT) and Aspartic acid Transaminase (AST)**

Fresh liver tissue was used for the ALT / AST assay. Normal saline was added at a weight-to-volume ratio of 1:9 (g: mL), and the homogenate was centrifuged at 2,500 rpm for 10 minutes. The supernatant was used for testing, and specific experimental steps followed the assay instructions [4]. The kits for the detection of ALT and AST were supplied by the Jiancheng Bioengineering Institute of Nanjing (Nanjing, China).

**Histological analyses**

Fresh liver tissue was fixed in 4% formalin solution for 24 hours, then dehydrated with a gradient of alcohols for 1 hour. The tissue was cleared with xylene, embedded in paraffin, and sectioned into 3 μm slices. The slices were dewaxed, rehydrated through a gradient of alcohols, stained with hematoxylin and eosin (H&E), then sealed with mounting gum. The stained sections were examined under a microscope.

**Total RNA extraction and quantitative real-time PCR (qRT-PCR)**

Liver tissue was suspended in ice-cold Trizol solution for 10 minutes at room temperature, then transferred to a 1.5 mL non-enzymatic Eppendorf tube. Total RNA was extracted using an RNA Easy Kit. qRT-PCR conditions were as follows: pre-denaturation at 94°C for 5 minutes, followed by 35 cycles of 94°C for 30 seconds, annealing at 50-60°C for 30 seconds, extension at 72°C for 30 seconds, and a final extension at 72°C for 10 minutes. Primer specificity was confirmed with melting curves, and standard curves were generated using reference cDNA to quantify the starting mRNA concentration in all samples [5]. The primer sequences for qRT-PCR were followed as Table S1.

**Western blot**

Protein samples were extracted from liver tissue. In brief, fresh liver tissue was ground and homogenized with cell lysis buffer (with 1:100 PMSF), then centrifuged at 13,000 rpm for 10 minutes. The supernatant was collected to determine protein concentration and divided into aliquots containing 80 µg of protein in 15 µL. The samples were boiled and stored at -20°C for subsequent western blot analysis. The specific operation method of immunoblotting should follow the previous description [2]. Color rendering of protein bands was performed using ECL Chemiluminescence Solution (Beijing Solarbio Science & Technology Co., Ltd., China). The protein band density was quantified by ImageJ software, and the results were normalized according to β-actin. The antibodies used are anti-β-actin (1:6000, Proteintech, 20536-1-AP), anti-p38 (1:1000, CST, 8690), anti-p-p38 (1:1000, CST, 4511), anti-p65 (1:1000, CST, 8242), anti-p-p65 (1:1000, CST, 3033), anti-SRBP1 (1:1000, CST, 95879), anti-FASN (1:1000, CST, 3180), anti-ACC (1:1000, CST, 3676S) and anti-p-ACC (1:1000, CST, 11818S).

**16S rDNA high throughput sequencing**

16S rDNA analysis was conducted using BMKCloud (www.biocloud.net). Total DNA was extracted from fecal samples (*n* = 6). After extraction, primers were designed based on conserved regions, with sequencing adaptors added to the primer ends for PCR amplification. The resulting products were purified, quantified, and normalized to create a sequencing library. Library quality was checked before sequencing on an Illumina HiSeq 2500. Reads were analyzed using QIIME for quantitative microbial ecology analysis. The alpha (α) and beta (β) diversity of gut microbiota in different samples was analyzed using the cloud platform. The abundance of taxonomic units at the phylum, family, and genus levels was counted for each type of flora in different groups.

**Fecal metabolite omics results**

Metabolomics analysis was also conducted using BMKCloud (www.biocloud.net). Fresh feces were collected from the colons of freshly euthanized mice, then immediately frozen and stored at -80°C. Metabolites were extracted from the samples via protein precipitation with organic solvents. Briefly, the fecal samples were quantified, homogenized, and centrifuged. The supernatants were used for liquid chromatograph-mass spectrometer (LC-MS) / MS analysis on an ultrahigh performance liquid chromatography (UHPLC) system (Vanquish; Thermo Fisher Scientific) with an ultra-performance liquid chromatography ethylene-bridge hybrid (UPLC BEH) Amide column coupled to a Q Extractive HF-X mass spectrometer (Orbitrap MS; Thermo), according to the manufacturer’s instructions. Samples were analyzed in both positive and negative ion modes. The acquired raw data was transformed into mzXML format. Subsequently, as previously described [6], it was processed with peak identification, peak alignment, peak extraction, retention time correction, and peak integration. Univariate and multivariate analyses were performed to identify differential metabolites between groups.

**Statistical analysis**

All statistical tests were carried out using GraphPad Prism 8.4.0 (GraphPad, CA, USA). Data are presented as means ± standard deviation (SD). Comparisons were made using Student's t-tests (general linear model) as appropriate. A *p*-value of less than 0.05 was considered statistically significant for this study.

# **Supplementary figures**

**FIGURE S1 High-Fat Diet (HFD) Induces Metabolic Disorders and Inflammation in Liver.**

(A) The protein expressions of Acetyl CoA carboxylase (ACC), p-ACC, fatty acid synthase (FASN), SRBP1, p65, p-p65, p38, p-p38 and β-actin. (B) Quantification of protein levels of p-ACC. (C) Quantification of protein levels of FASN. (D) Quantification of protein levels of SRBP1. (E) Quantification of protein levels of p-p65. (F) Quantification of protein levels of p-p38. Data are presented as means ± standard deviation (SD) (*n* = 3). Statistical significance is indicated as follows: *^*^p* < 0.05, *^**^p* < 0.01, *^***^p* < 0.001, *^****^p* < 0.0001.

**FIGURE S2 The Impact of HFD on the Intestinal Microbiota.**

(A) Shannon curves. Each curve represents a sample, with the control group shown in yellow and the HFD group in blue. (B) Species accumulation curves. The red box lines represent accumulation curves, while the green box lines represent total volume curves. (C) Principal coordinates analysis (pCoA). Control and HFD groups are marked in yellow and blue, respectively; each point represents an individual sample. (D) unweighted pair-group method with arithmetic means (UPGMA) analysis. Samples from the control and HFD groups are shown in yellow and blue, respectively. Shorter branch lengths indicate greater similarity in species composition between samples. (E) Heat map analysis. The color gradient from blue to red represents increasing similarity among samples. (F) Venn diagram. The control group is shown in blue, and the HFD group in yellow; the numbers in the overlapping section indicate the number of shared features between the two groups.

**FIGURE S3 Analysis of Gut Microbiota Composition.**

(A-D) Histogram of aerobic, anaerobic, Gram-negative, and Gram-positive species analyzed with Bug Base. This comparison demonstrates high and low species diversity, abundance similarity, and dominant species for each sample based on the proportion of each color block.

**FIGURE S4 Non-Targeted Metabolomic Analysis of Mouse Gut Microbiota.**

(A) Principal component analysis (PCA) of total metabolites. Red dots represent control samples, and blue triangles represent HFD groups. Different symbols correspond to different samples. (B) Clustering analysis of differential metabolites in control and HFD groups. Red regions indicate differential metabolites up-regulated in HFD, while green regions indicate those down-regulated. (C) Inter-sample correlation assessment. This assesses the correlation between samples within groups. A higher intra-group correlation coefficient relative to inter-group indicates reliable differential metabolites. The Spearman rank correlation was used, and the correlation coefficient r^2^ closer to 1 suggests stronger correlation between replicates. (D) PCA analysis of differential metabolites. Red dots represent control samples, and blue triangles represent HFD groups. (E) Clustering analysis of differential metabolites in control and HFD groups. (F) Volcano plot of differential metabolites. Black dots represent unchanged metabolites, red dots represent those up-regulated in HFD, and green dots represent those down-regulated. (G) Human metabolome database (HMDB) classification plot of differential metabolites. This plot displays the top 20 metabolic classifications, sorted from largest to smallest. (H) Kyoto encyclopedia of genes and genomes (KEGG) enrichment plot of differential metabolites. The horizontal axis represents the ratio of differential metabolites with pathway annotations to all differential metabolites in a pathway.

# **Supplementary tables**

**Table S1** Primers for qRT-PCR

| Gene | Sequences (5'-3') |
| --- | --- |
| IL-1β | (F) GTTCCCATTAGACAACTGCACTACAG |
|  | (R) GTCGTTGCTTGGTTCTCCTTGTAH |
| IL-6 | (F) CCAGAAACCGCTATGAAGTTCC |
|  | (R) GTTGGGAGTGGTATCCTCTGTGAF |
| TNF-α | (F) CCCCAAAGGGATGAGAAGTTCP |
|  | (R) CCTCCACTTGGTGGTTTGCT |
| Inos | (F) GAACTGTAGCACAGCACAGGAAATH |
|  | (R) CGTACCGGATGAGCTGTGAAT |
| Cox2 | (F) CAGTTTATGTTGTCTGTCCAGAGTTTCH |
|  | (R) CCAGCACTTCACCCATCAGTTF |

**References:**

1. Alvarez, Daniela, Macarena Ortiz, Gabriel Valdebenito, Nicolas Crisosto, Barbara Echiburu, Rodrigo Valenzuela, Alejandra Espinosa, Manuel Maliqueo. 2023. "Effects of a High-Fat Diet and Docosahexaenoic Acid during Pregnancy on Fatty Acid Composition in the Fetal Livers of Mice." *Nutrients* 15: https://doi.org/10.3390/nu15214696

2. Liu, Shu, Shoupeng Fu, Yuhang Jin, Ruiqi Geng, Yuhang Li, Yufei Zhang, Juxiong Liu, Wenjin Guo. 2023. "Tartary buckwheat flavonoids alleviates high-fat diet induced kidney fibrosis in mice by inhibiting MAPK and TGF-beta1/Smad signaling pathway." *Chem Biol Interact* 379: 110533. https://doi.org/10.1016/j.cbi.2023.110533

3. Gong, Qian, Yanwei Li, He Ma, Wenjin Guo, Xingchi Kan, Dianwen Xu, Juxiong Liu, Shoupeng Fu. 2018. "Peiminine Protects against Lipopolysaccharide-Induced Mastitis by Inhibiting the AKT/NF-kappaB, ERK1/2 and p38 Signaling Pathways." *Int J Mol Sci* 19: https://doi.org/10.3390/ijms19092637

4. Ran, Xin, Guiqiu Hu, Fuding He, Kefei Li, Feng Li, Dianwen Xu, Juxiong Liu, Shoupeng Fu. 2022. "Phytic Acid Improves Hepatic Steatosis, Inflammation, and Oxidative Stress in High-Fat Diet (HFD)-Fed Mice by Modulating the Gut-Liver Axis." *J Agric Food Chem* 70: 11401-11411. https://doi.org/10.1021/acs.jafc.2c04406

5. Bai, Juli, Christopher Cervantes, Juan Liu, Sijia He, Haiyan Zhou, Bilin Zhang, Huan Cai, et al. 2017. "DsbA-L prevents obesity-induced inflammation and insulin resistance by suppressing the mtDNA release-activated cGAS-cGAMP-STING pathway." *Proc Natl Acad Sci U S A* 114: 12196-12201. https://doi.org/10.1073/pnas.1708744114

6. Tao, Zixin, Yun Chen, Fang He, Jiawei Tang, Limei Zhan, Haoyue Hu, Ziling Ding, et al. 2023. "Alterations in the Gut Microbiome and Metabolisms in Pregnancies with Fetal Growth Restriction." *Microbiol Spectr* 11: e0007623. https://doi.org/10.1128/spectrum.00076-23
